# 基于亲水相互作用的超高效液相色谱-三重四极杆质谱法测定不同成熟阶段草莓中18种游离氨基酸

**DOI:** 10.3724/SP.J.1123.2024.04017

**Published:** 2025-04-08

**Authors:** Min JU, Yuming SONG, Jinfeng ZHAO, Yuming SUN, Lina ZHOU, Qingxin YIN, Chen WANG, Rui CAI, Qiang XU, Huihui WAN

**Affiliations:** 1.大连理工大学分析测试中心,辽宁 大连 116024; 1. Instrumental Analysis Center, Dalian University of Technology, Dalian 116024, China; 2.大连理工大学化工学院,辽宁 大连 116024; 2. School of Chemical Engineering, Dalian University of Technology, Dalian 116024, China

**Keywords:** 超高效液相色谱, 亲水相互作用色谱, 串联质谱, 游离氨基酸, 草莓, ultra performance liquid chromatography (UPLC), hydrophilic interaction chromatography (HILIC), tandem mass spectrometry (MS/MS), free amino acids, strawberry

## Abstract

不同成熟时期草莓中游离氨基酸的组成和含量测定对草莓的品质评价和营养研究具有重要意义。本研究建立了基于亲水相互作用的超高效液相色谱-串联质谱技术快速直接测定不同成熟阶段草莓中18种游离氨基酸的分析方法。将经过研磨后的草莓样品用水提取并通过离心和膜过滤后,进行液相色谱-串联质谱分析。采用ACQUITY UPLC Glycan BEH Amide亲水相互作用液相色谱柱(150 mm×2.1 mm, 1.7 μm),以含有5 mmol/L甲酸铵和0.1%甲酸的乙腈水溶液为流动相进行梯度洗脱。采用三重四极杆质谱仪,在电喷雾离子源正离子扫描模式下,以基质匹配标准曲线法对氨基酸定量分析。结果表明,该方法在0.5~40.0μmol/L范围内线性关系良好(*r*^2^≥0.992)。方法的日内精密度为1.0%~14.8%,日间精密度为3.6%~17.6%;检出限在50~250 nmol/L范围内。在3个添加水平下,草莓基质中样品加标回收率为75.0%~114.6%,相对标准偏差为0.3%~13.5%。将建立的定量分析方法用于5个不同成熟时期草莓中氨基酸含量的测定,经统计分析后共筛选出7种差异性游离氨基酸(苯丙氨酸、异亮氨酸、谷氨酰胺、4-氨基丁酸、精氨酸、谷氨酸和天冬氨酸)。该方法快速、准确,具有良好的重复性和稳定性,可对草莓中游离氨基酸组成进行定量检测。

## Abstract (en)

The determination of free amino acids is important for quality evaluation and nutritional studies of strawberries. In this study, an ultra performance liquid chromatography-tandem mass spectrometry (UPLC-MS/MS) method using hydrophilic interaction chromatographic column was established for the simultaneous determination of 18 free amino acids in strawberries, and the pretreatment, chromatography, and mass spectrometry conditions were optimized. Specific pretreatment processes include grinding, extraction with water, centrifugation, and membrane filtration. The treated samples were then analyzed by hydrophilic interaction liquid chromatography (HILIC)-tandem mass spectrometry. The separation was performed on an ACQUITY UPLC Glycan BEH Amide column (150 mm×2.1 mm, 1.7 μm), with a water-acetonitrile system containing 5 mmol/L ammonium formate and 0.1% (v/v) formic acid as the mobile phase for gradient elution. A triple quadrupole mass spectrometer was used in positive-ion electrospray ionization (ESI) scanning mode, with target amino acids quantified using the matrix-matched standard-curve method. Eighteen free amino acids were determined with good linearities in the range of 0.5-40.0 μmol/L, along with *r*^2^ greater than 0.992. The intra-day and inter-day precisions of the method were 1.0%-14.8% and 3.6%-17.6%, respectively. The limits of detection (LODs) were in the range of 50-250 nmol/L. The recoveries of the 18 amino acids were 75.0%-114.6% with relative standard deviations (RSDs) of 0.3%-13.5%. The contents of free amino acids in strawberries at different ripening stages were statistically analyzed, and a total of seven differentiated free amino acids (phenylalanine, isoleucine, glutamine, 4-aminobutyric acid, arginine, glutamic acid, and aspartic acid) were statistically screened. The method is rapid, accurate, reproducible and stable, and can quantitatively determine the content of amino acids in strawberries.

草莓是一种蔷薇科(Rosaceae)草莓属(*Fragaria*)的多年生草本植物^[1,2]^,其果实有着较高的营养价值和经济价值^[3,4]^。草莓中含量丰富的氨基酸与草莓品质、色泽度和香气有着密切的关联^[5]^。草莓中游离的脯氨酸、天冬氨酸和缬氨酸能够与花青素发生共色素沉着效应,可显著增加花青素的热稳定性^[5⇓-7]^,从而增加草莓的抗氧化能力。此外,草莓中丰富的氨基酸具有抗突变、降低血糖和减少冠心病等功效^[4,5]^。

氨基酸的测定方法包括离子交换色谱法^[8]^、毛细管电泳法^[9]^、气相色谱法^[10]^、高效液相色谱法(HPLC)^[11,12]^、液相色谱-质谱联用法^[13⇓⇓-16]^等。离子交换色谱法包括阴离子交换色谱法与阳离子交换色谱法,阴离子交换色谱法通常与积分脉冲安培检测器联用,缺点是分离和平衡时间较长^[17]^。氨基酸自动分析仪采用阳离子交换色谱与茚三酮或荧光胺柱后衍生结合的方法对氨基酸进行分离后,使用紫外可见光或荧光等检测器对氨基酸衍生物进行测定^[14,18⇓-20]^。毛细管电泳法可直接测定氨基酸,但缺点是重复性较差^[18,21,22]^。由于氨基酸极性较大且不易挥发,需要将其衍生化为极性较低的挥发性衍生物才能利用气相色谱法进行检测^[23,24]^。HPLC是分析化学中最常用的色谱技术,由于大多数氨基酸无生色基团,需将其通过柱前或柱后衍生化为具有生色团的衍生物才能使用紫外或荧光等检测器对其进行测定。常用的衍生化试剂包括邻苯二甲醛(*o*-phthalaldehyde, OPA)^[25]^、6-氨基喹啉基-*N*-羟基琥珀酰亚胺-氨基甲酸酯(6-aminoquinolyl-*N*-hydroxy-succinimidyl carbamate, AQC)^[14,26]^、异硫氰酸苯酯(phenyl isothiocyanate, PITC)^[27]^和丹磺酰氯(dansyl chloride, DNS-Cl)^[28]^等。液相色谱-质谱联用法中常用反相模式,但反相模式无法有效保留极性较大的氨基酸,需对氨基酸柱前衍生化,以增加衍生物在色谱柱上的保留^[29,30]^。与反相模式相比,采用亲水相互作用色谱柱的液相色谱分离模式,基于极性化合物与极性固定相和含水流动相的多种相互作用(如氢键、分配、吸附和离子交换等),能有效保留和分离极性较大的氨基酸等目标化合物,适用于复杂样品中极性目标化合物的直接测定^[18]^。随着质谱检测器的发展,通过质荷比(*m/z*)进一步对化合物进行分离,在一定程度上解决了色谱峰不能完全分离的问题^[18,31,32]^。Wang等^[5]^通过非靶向代谢组学分析的方法对草莓中的代谢物进行筛选;Bingöl等^[7]^研究了天冬氨酸、脯氨酸和缬氨酸对草莓汁中花青素的共着色作用;Huang等^[33]^利用紫外激发荧光成像技术监测草莓在储存过程中的质量变化,但目前关于不同时期草莓中游离氨基酸含量的测定方法尚未见报道。

草莓中游离氨基酸组成和含量的测定对草莓的品质评价和营养研究具有重要意义。本研究采用亲水相互作用色谱柱,通过对前处理和色谱-质谱条件进行优化,使用电喷雾离子源(ESI)在正离子扫描模式下,建立了超高效液相色谱-三重四极杆质谱(UPLC-MS/MS)技术直接测定草莓中游离氨基酸的方法,并进行了详细的方法验证,同时对不同成熟时期草莓样品中游离氨基酸的含量进行了统计分析。

## 1 实验部分

### 1.1 仪器、材料与试剂

ACQUITY UPLC I-Class液相色谱系统(美国Waters公司); QTRAP 6500+三重四极杆质谱仪(美国SCIEX公司); Analyst 1.7.2软件用于仪器控制;SCIEX OS-Q 2.2.0软件用于数据处理;Milli-Q Direct8超纯水系统(德国Merck Millipore公司); Tiss-Basic多样品组织研磨机(上海净信实业发展有限公司);尼龙滤膜(0.2 μm,美国Thermo Scientific公司)

18 种氨基酸标准品(纯度均>96%):丙氨酸(alanine, Ala)、4-氨基丁酸(4-aminobutyric acid, GABA)、谷氨酸(glutamic acid, Glu)、蛋氨酸(methionine, Met)、脯氨酸(proline, Pro)、丝氨酸(serine, Ser)、精氨酸(arginine, Arg)、天冬酰胺(asparagine, Asn)、天冬氨酸(aspartic acid, Asp)、谷氨酰胺(glutamine, Gln)、组氨酸(histidine, His)、异亮氨酸(isoleucine, Ile)、亮氨酸(leucine, Leu)、赖氨酸(lysine, Lys)、苯丙氨酸(phenylalanine, Phe)、苏氨酸(threonine, Thr)、酪氨酸(tyrosine, Tyr)、缬氨酸(valine, Val)购自坛墨质检科技股份有限公司;乙腈(色谱纯,赛默飞世尔科技有限公司);甲酸(色谱纯,北京迪科马科技有限公司);甲酸铵(色谱纯,德国Honeywell公司)。不同成熟阶段的草莓样品取自大连市岔鞍村草莓种植园。

### 1.2 样品前处理与溶液配制

#### 1.2.1 样品前处理

用研磨机将草莓样品研磨1 min后,准确称取草莓匀浆0.625 g于25 mL容量瓶中,使用纯水定容后取1 mL于离心管中,以12000 r/min离心5 min,取离心后的上清液100 μL于离心管中,加入400 μL甲醇纯化、涡旋后再次以12000 r/min离心5 min,取上清液,经0.2 μm滤膜过滤,供色谱-质谱分析。

#### 1.2.2 标准溶液的配制

氨基酸混合标准溶液(1 mmol/L)用50%乙腈水溶液逐级稀释成浓度分别为0.5、1.0、5.0、10.0、20.0、25.0、40.0 μmol/L的一系列混合标准溶液。

取80 μL处理后的样品溶液,加入适量1 mmol/L氨基酸混合标准溶液,配制成一系列的氨基酸混合基质匹配标准溶液,浓度分别为0.5、1.0、5.0、10.0、20.0、25.0、40.0 μmol/L。由于草莓中氨基酸含量差异很大,其中谷氨酰胺的含量较高,因此在测定谷氨酰胺时将处理后的样品溶液稀释至原浓度的1/50后再进行相应浓度的基质匹配标准溶液的配制。

### 1.3 分析条件

#### 1.3.1 色谱条件

ACQUITY UPLC Glycan BEH Amide色谱柱(150 mm×2.1 mm, 1.7 μm,美国Waters公司);柱温:30 ℃;流动相A为10%乙腈水溶液(含5 mmol/L甲酸铵和0.1%甲酸),流动相B为90%乙腈水溶液(含5 mmol/L甲酸铵和0.1%甲酸);梯度洗脱程序:0~3 min, 100%B~80%B; 3~12 min, 80%B~40%B; 12~15 min, 40%B; 15~16 min, 40%B~100%B; 16~26 min, 100%B。流速:0.3 mL/min;进样量:1 μL。

#### 1.3.2 质谱条件

离子源:ESI,正离子扫描;多反应监测模式(multiple reaction monitoring, MRM);离子源温度:500 ℃;离子化电压:5500 V;喷雾气压力:0.345 MPa;气帘气压力:0.276 MPa;辅助加热气压力:0.379 MPa。

#### 1.3.3 数据分析

使用Excel进行*t*检验计算,检验氨基酸在两组间差异的显著性,当*P*≤0.05时认为具有统计学差异,*P*≤0.01认为差异显著;通过metaboAnalyst 6.0(https://www.metaboanalyst.ca/)对偏最小二乘-判别分析(partial least squares-discriminant analysis, PLS-DA)模型进行分析和验证,筛选草莓在不同成熟时期的差异性氨基酸。

## 2 结果与讨论

### 2.1 质谱条件优化

采用单标单针进样方式,对氨基酸的质谱条件进行优化,因分析物在正离子模式测得的质谱信号强度均高于负离子模式,因此选择在电喷雾正离子模式下进行全扫描。将稳定性好且强度高的[M+H]^+^作为母离子,同时选择合适的碎片离子作为子离子,与母离子组成离子对后,优化碰撞能量(collision energy, CE)和去簇电压(declustering potential, DP),表1为18种氨基酸经优化后的质谱采集参数。对氨基酸混合标准溶液进行检测,得到的提取离子流色谱图见图1。分析结果与Vilches等^[34]^报道相一致,异亮氨酸和赖氨酸母离子出现同时丢失-CH_2_O_2_和-NH_3_基团的碎片情况,除精氨酸、天冬酰胺、谷氨酸、4-氨基丁酸、蛋氨酸和谷氨酰胺外,其他氨基酸的母离子碎裂为丢失-CH_2_O_2_基团的特征碎片。赖氨酸与谷氨酰胺具有相同的离子对,但是保留时间分别为7.52 min和6.20 min,因此可以通过保留时间区分这两种氨基酸。

**表1:** 18种氨基酸的质谱参数

No.	Compound	Retention time/min	Parent ion (*m/z*)	Product ions (*m/z*)	Declustering potential/V	Collison energies/eV
1	Ala	5.68	90.0	44.0^*^	20	12
2	Arg	7.37	175.2	70.2^*^, 116.1	40	27, 20
3	Asn	6.37	133.1	74.2^*^, 87.2	40	20, 12
4	Ile	4.64	132.1	69.2^*^, 86.1	40	24, 14
5	Glu	6.15	148.1	83.9^*^, 102.0	30	20, 15
6	His	7.49	156.1	110.0^*^, 83.1	30	20, 32
7	GABA	5.08	104.0	87.0^*^, 69.4	40	13, 20
8	Asp	6.75	133.9	88.0^*^, 74.2	30	14, 19
9	Leu	4.47	132.1	86.2^*^, 104.1	30	14, 11
10	Pro	5.12	116.1	70.0^*^, 43.3	30	20, 38
11	Thr	5.91	119.9	74.0^*^, 56.2	20	14, 20
12	Phe	4.36	166.2	120.2^*^, 102.9	30	17, 38
13	Tyr	4.98	182.2	136.1^*^, 165.1	20	19, 13
14	Ser	6.28	106.1	60.2^*^, 42.2	20	13, 25
15	Val	5.04	118.1	72.3^*^, 55.1	30	15, 27
16	Met	4.77	150.0	133.2^*^, 104.2	30	13, 13
17	Gln	6.20	147.1	130.1^*^, 84.1	40	13, 22
18	Lys	7.52	147.1	84.1^*^, 130.1	30	21, 13

*Quantitative ion.

**图1:**
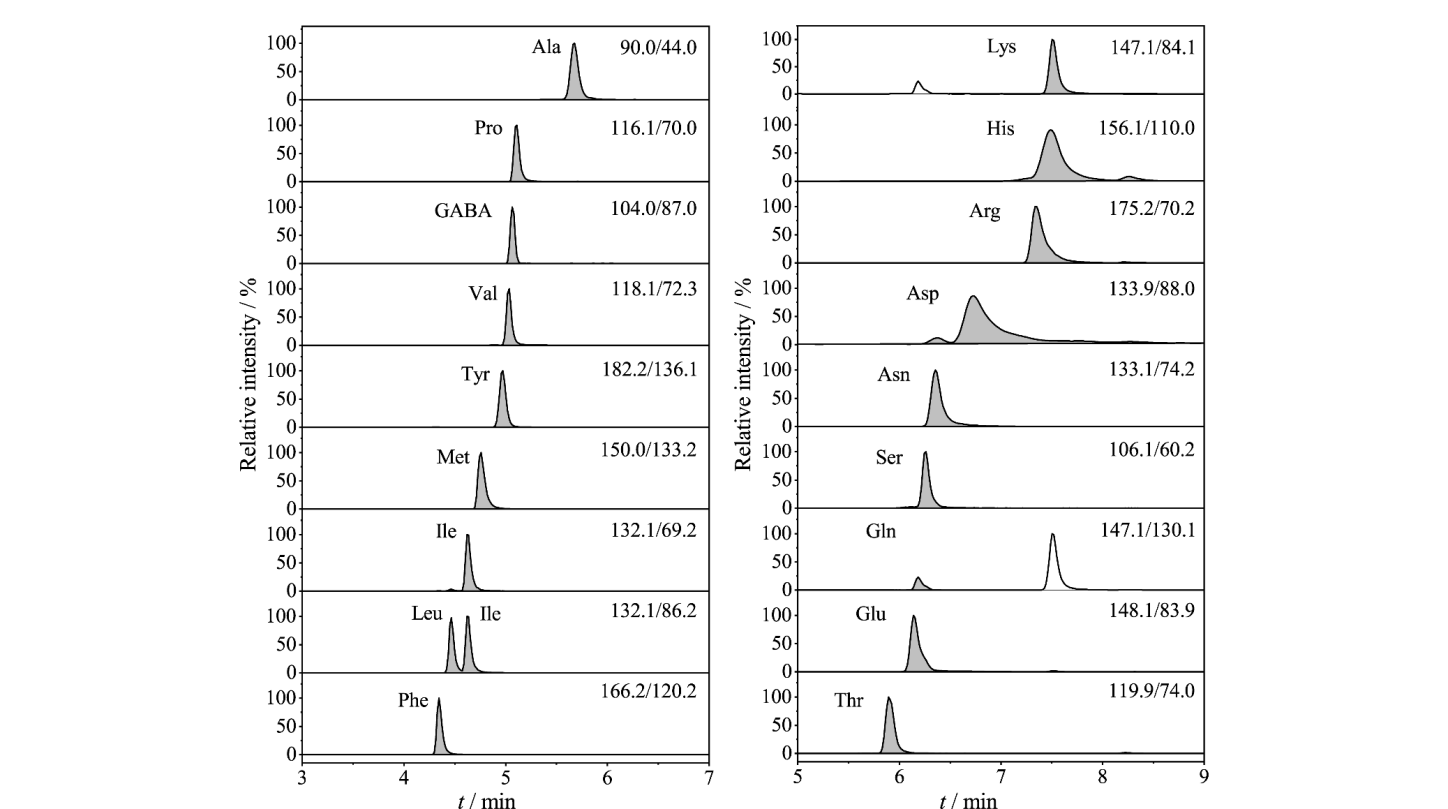
18种游离氨基酸的提取离子流色谱图

### 2.2 样品前处理条件的优化

虽然不同成熟时期的草莓本身含水量会有一些差异^[35,36]^,但游离氨基酸也会以游离态存在于自由水中,无论是烘干还是其他降低含水量的前处理,都可能导致游离氨基酸含量的变化,尤其是烘干过程部分氨基酸会发生分解^[37⇓⇓-40]^。综合以上考虑,我们在前处理时直接对新鲜的草莓样品进行提取。将新鲜草莓表面的灰尘用水冲洗干净,用无尘纸擦拭并充分晾干后用于后续的样品前处理优化、方法验证、样品测试等后续实验操作。

#### 2.2.1 提取液组成的优化

氨基酸的极性比较大,文献中报道的氨基酸的提取溶剂包括水^[41]^、盐酸^[27,42]^以及水-乙醇混合液^[43]^等。在实验过程中考察了不同比例的水-乙醇体系对草莓中游离氨基酸提取效果的影响,比较了乙醇体积分数分别为0、25%、50%、75%、100%时对草莓中18种游离氨基酸的提取效果,结果如图2a所示。除谷氨酸、酪氨酸和谷氨酰胺外,水对游离氨基酸的提取效果均优于水-乙醇混合液,因此选用水作为提取液。酸性环境也会影响氨基酸的提取效果,因此在水中分别加入不同体积分数的甲酸(0、0.05%、0.1%、0.2%和0.5%)作为提取溶剂,提取结果如图2b所示,在水中添加不同比例的甲酸对草莓中游离氨基酸的提取效果影响不大。此外,酸性环境可能会造成多肽和蛋白质水解,水解产物对游离氨基酸的测定容易形成干扰^[44]^,因此实验选择纯水作为提取溶剂。

**图2:**
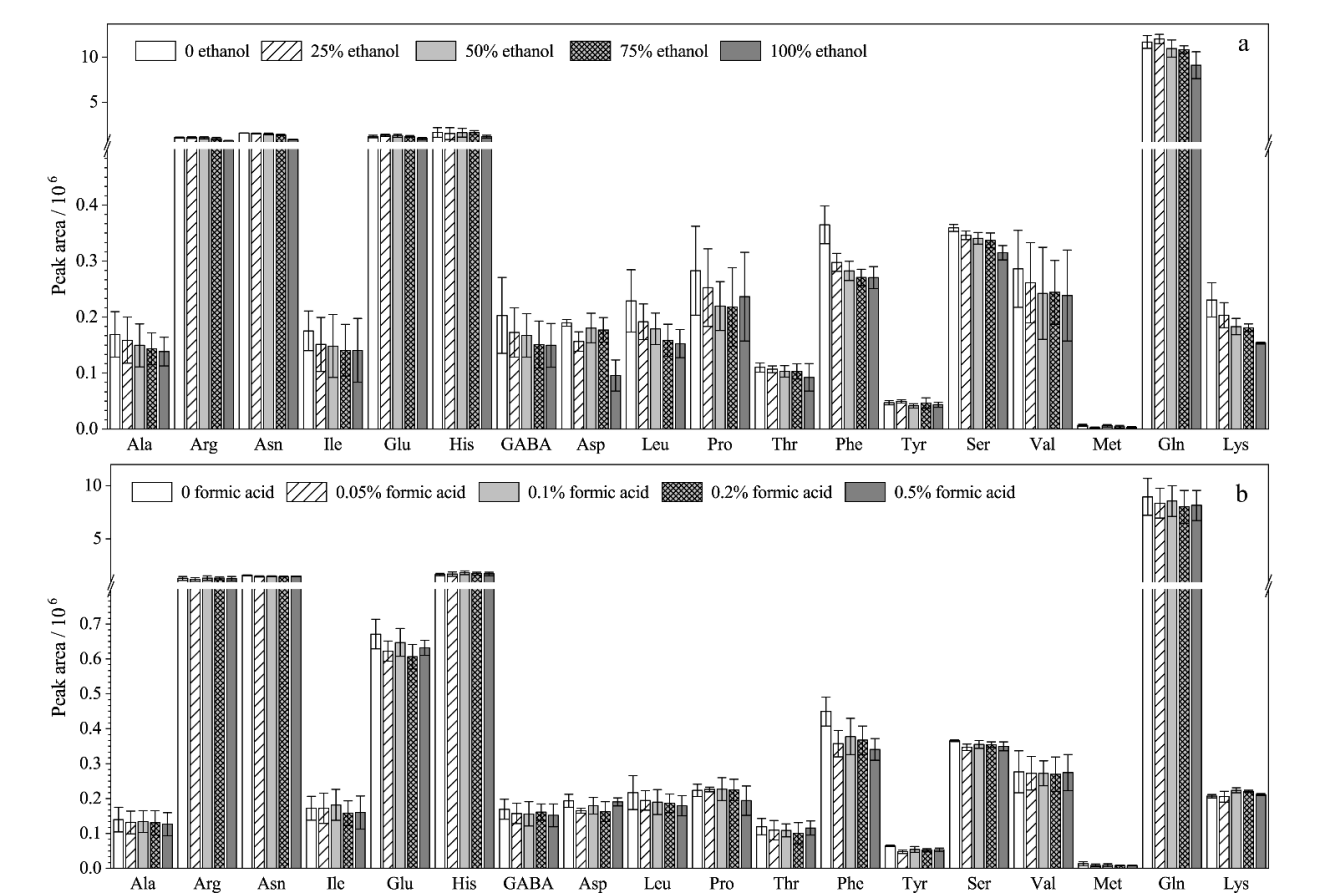
提取溶剂对草莓中游离氨基酸提取效率的影响(*n*=3)

#### 2.2.2 超声提取时间的优化

以水作为提取溶剂,比较超声提取时间分别为0、5、10、20 min对氨基酸提取效率的影响。如图3所示,除精氨酸、组氨酸和丝氨酸在10 min时峰面积略高于其他超声提取时间的峰面积外,不同的超声提取时间对于同一氨基酸峰面积的影响基本无差别。因此,为简化前处理操作,缩短前处理时间并进行快速测定,本实验选择不采用超声手段。氨基酸的色谱峰面积与超声提取时间没有出现明显的正比例关系,分析原因这可能归因于大多数氨基酸具有良好的水溶性及稳定性^[45]^。

**图3:**
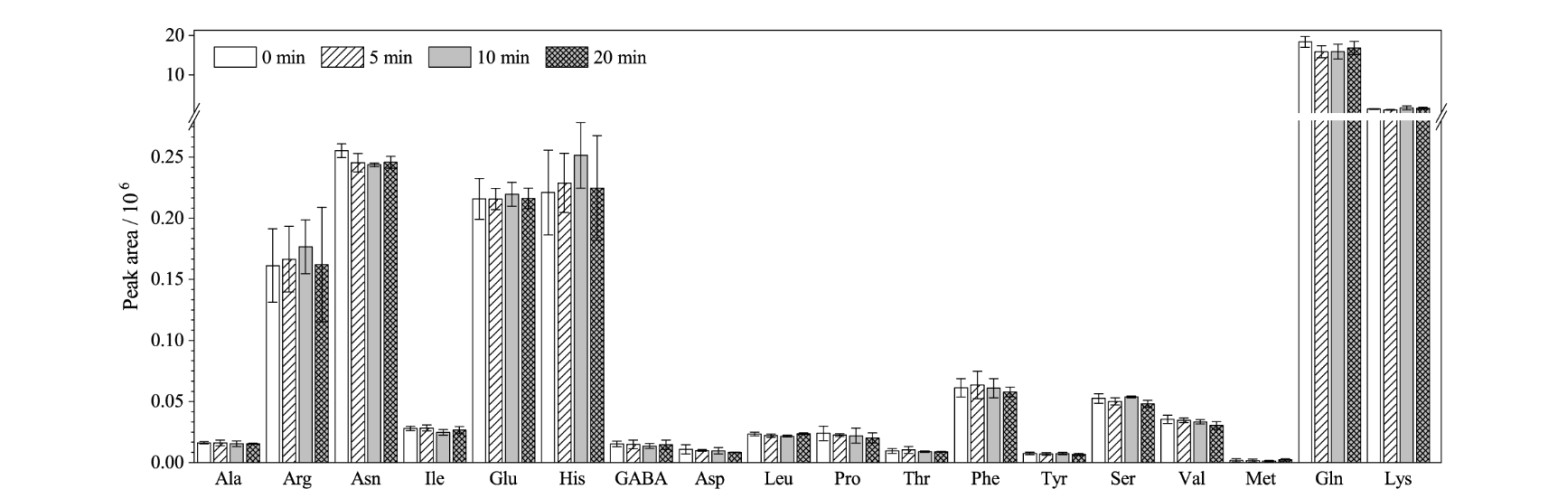
超声时间对草莓中游离氨基酸提取效率的影响(*n*=3)

### 2.3 方法学验证

#### 2.3.1 线性范围、检出限和定量限

与溶剂相比,草莓基质复杂,除含有氨基酸外,还含有一些干扰氨基酸测定的其他物质,这种干扰称为基质效应(matrix effect, ME)。从表2可以看出,采用基质匹配标准曲线与溶剂标准曲线斜率的比值计算基质效应,大多数氨基酸基质效应在93.86%~110.48%范围内,说明基质效应较弱。但是精氨酸、组氨酸、天冬氨酸和谷氨酰胺的基质效应分别为118.26%、118.27%、114.70%和113.91%,说明存在一定的基质增强效应。为降低草莓中游离氨基酸测定时基质带来的干扰,实验采用基质加标的方法绘制基质匹配标准曲线进行定量分析。

**表2:** 18种氨基酸的线性范围、回归方程、相关系数、检出限和基质效应

No.	Compound	Linear range/(μmol/L)	Linear equation	*r*^2^	LOD/(nmol/L)	ME/%
1	Ala	0.5-40.0	*y*=2325.5*x*+3762.8	0.996	100	105.40
2	Arg	0.5-40.0	*y*=413946.0*x*+277758.0	0.993	50	118.26
3	Asn	0.5-40.0	*y*=9726.9*x*+169119.0	0.997	100	101.83
4	Ile	0.5-40.0	*y*=52658.1*x*+5822.4	0.999	50	108.77
5	Glu	0.5-40.0	*y*=31522.2*x*+36703.0	0.999	100	93.86
6	His	0.5-40.0	*y*=218849.0*x*+49215.2	0.998	100	118.27
7	GABA	0.5-40.0	*y*=10913.9*x*+15204.0	0.998	100	95.25
8	Asp	0.5-40.0	*y*=6251.4*x*+6305.6	0.998	100	114.70
9	Leu	0.5-40.0	*y*=43221.3*x*+3021.3	0.999	50	96.87
10	Pro	0.5-40.0	*y*=57499.8*x*+12560.5	0.994	100	102.96
11	Thr	0.5-40.0	*y*=9086.8*x*+4237.1	0.997	250	100.83
12	Phe	0.5-40.0	*y*=180570.0*x*-8084.2	0.999	100	110.48
13	Tyr	0.5-40.0	*y*=32588.4*x*+1813.2	0.999	100	101.57
14	Ser	0.5-40.0	*y*=4438.0*x*+12601.9	0.998	50	100.29
15	Val	0.5-40.0	*y*=9584.1*x*+6998.3	0.997	100	104.75
16	Met	0.5-40.0	*y*=12958.6*x*+1561.3	0.997	100	95.76
17	Gln	0.5-40.0	*y*=2419.3*x*+56090.2	0.999	100	113.91
18	Lys	0.5-40.0	*y*=88302.4*x*+48543.0	0.992	100	109.18

*y*: peak area; *x*: concentration, μmol/L.

从图3的结果可以看出,草莓中谷氨酰胺在草莓中的含量远高于其他氨基酸,因此将处理后的样品溶液稀释至原浓度的1/50,测定谷氨酰胺。以18种氨基酸的浓度为横坐标*x*,以定量离子的峰面积为纵坐标*y*,计算回归方程。如表2所示,18种目标氨基酸在0.5~40.0 μmol/L范围内线性关系良好,相关系数(*r*^2^)≥0.992。以信噪比(*S/N*)≥3对应的氨基酸混合标准溶液浓度作为氨基酸的检出限(LOD),测得18种氨基酸的检出限范围为50~250 nmol/L。以*S/N*≥10对应的氨基酸混合标准溶液浓度作为氨基酸的定量限(LOQ),其中精氨酸、异亮氨酸、亮氨酸和丝氨酸的定量限为100 nmol/L;天冬酰胺、谷氨酸、天冬氨酸、蛋氨酸和赖氨酸的定量限为250 nmol/L;其他9种氨基酸的定量限为500 nmol/L。

#### 2.3.2 加标回收率、日内和日间精密度

采用样品加标的方法,进行加标回收试验。谷氨酰胺的加标水平为5.0、10.0和20.0 μmol/L,其他氨基酸为1.0、10.0和25.0 μmol/L,每个加标水平取6次平行样本进行测定。实验结果如表3所示,样品加标回收率为75.0%~114.6%,相对标准偏差(RSD)为0.3%~13.5%。

**表3:** 18种氨基酸的加标回收率和精密度(*n*=6)

No.	AA	Added/ (μmol/L)	Recovery/ %	RSD/ %	Intra-day precision/%	Inter-day precision/%	No.	AA	Added/ (μmol/L)	Recovery/ %	RSD/ %	Intra-day precision/%	Inter-day precision/%
1	Ala	1.0	88.4	11.8	4.9	11.0	10	Pro	1.0	93.2	3.2	7.8	3.9
		10.0	97.6	1.2					10.0	101.1	0.8		
		25.0	96.1	3.7					25.0	106.1	2.1		
2	Arg	1.0	94.6	6.2	4.7	3.6	11	Thr	1.0	97.3	9.0	4.6	6.1
		10.0	97.9	2.1					10.0	92.6	6.6		
		25.0	100.3	3.0					25.0	106.6	3.4		
3	Asn	1.0	82.0	13.1	1.0	7.2	12	Phe	1.0	111.1	1.8	14.8	6.5
		10.0	78.7	3.8					10.0	102.2	1.0		
		25.0	79.6	2.9					25.0	114.6	1.8		
4	Ile	1.0	87.7	2.4	5.9	6.1	13	Tyr	1.0	97.5	4.6	4.6	10.0
		10.0	87.4	4.9					10.0	90.2	2.6		
		25.0	107.2	5.1					25.0	107.8	2.5		
5	Glu	1.0	92.3	4.3	3.7	9.3	14	Ser	1.0	75.0	11.9	2.9	16.8
		10.0	96.8	2.3					10.0	83.1	3.7		
		25.0	98.0	1.4					25.0	82.6	2.9		
6	His	1.0	103.0	2.3	1.9	5.0	15	Val	1.0	94.5	3.7	6.6	4.8
		10.0	99.1	0.3					10.0	85.7	1.2		
		25.0	101.1	1.7					25.0	96.2	2.3		
7	GABA	1.0	93.0	4.4	8.3	4.7	16	Met	1.0	93.0	4.8	10.1	17.6
		10.0	82.1	5.4					10.0	88.8	3.5		
		25.0	102.2	3.0					25.0	106.2	3.2		
8	Asp	1.0	91.1	13.5	4.5	6.9	17	Gln	5.0	81.7	7.6	2.2	17.5
		10.0	85.1	6.2					10.0	95.2	6.5		
		25.0	97.4	7.7					20.0	97.2	4.2		
9	Leu	1.0	88.5	2.7	6.7	4.8	18	Lys	1.0	97.1	4.9	1.8	5.3
		10.0	88.3	4.2					10.0	96.3	1.7		
		25.0	102.0	7.0					25.0	101.6	3.1		

在同一天内每隔2 h对氨基酸混合标准溶液测定一次,重复测定6次,确定方法的日内精密度;连续6天对同一浓度的氨基酸混合标准溶液进行检测,确定方法的日间精密度,结果以RSD表示。实验结果如表3所示,方法的日内精密度为1.0%~14.8%,日间精密度为3.6%~17.6%。

#### 2.3.3 与文献结果比较

与文献[15⇓-17,20,22,24,30⇓-32]中报道的氨基酸测定方法相比(表4),本文方法的回收率和检出限与文献相当。同时,本实验建立的方法可对草莓中的游离氨基酸进行直接测定,有效避免了衍生化过程所带来的衍生产物不稳定和影响测定的干扰物等问题。

**表4:** 文献中的氨基酸分析数据与本实验结果比较

No.	Method	Linear range/(μmoL/L)	Sample	Analysis time/min	LOD/(nmoL/L)	Recovery/%	Ref.
1	IC-IPAD	0.2-260.7	navel orange	74	42-1449	72.5-130.5	[17]
2	AAA	0.4-88.5	shellfish	53	70-270	86.4-102.4	[20]
3	CE-in-UV	20.0-600.0	tea	14	1700-4500	83.0-106.0	[22]
4	GC-FID	1.0-2665.5	citrus	28	121-2380	72.8-133.0	[24]
5	UPLC-PDA	5.0-1000.0	fish eggs	10	940-4040	75.4-107.3	[30]
6	UPLC-HRMS	41.5-2631.6	eriocheir sinensis	12	1250-3998	78.4-105.3	[31]
7	UPLC-MS/MS	0.1-469.8	ziziphus jujubaby	12	2-642	93.5-103.6	[32]
8	UPLC-MS/MS	0.0-6.0	sweet potato	19	5-296	60.0-91.0	[15]
9	UPLC-MS/MS	1.0-50.0	zanthoxylum bungeanum	8	3-78	60.4-120.4	[16]
10	UPLC-MS/MS	0.5-40.0	strawberry	26	50-250	75.0-114.6	this work

IC-IPAD: anion exchange chromatography with integrated pulsed amperometric detection; AAA: automatic amino acid analyzer; CE-in-UV: capillary electrophoretic separation and indirect ultraviolet detection; GC-FID: gas chromatography with hydrogen flame ionization detector; PDA: photo-diode array detector; HRMS: high resolution mass spectrometry; UPLC-MS/MS: ultra performance liquid chromatography with triple quadrupole mass spectrometry.

### 2.4 实际样品检测

采用上述方法测定不同成熟时期草莓中游离氨基酸组分的含量,每个成熟时期各取6份平行样品进行测定。除脯氨酸含量低于定量限,不能准确计算含量外,其余17种游离氨基酸在草莓不同成熟期的含量如图4所示。其中含量最多的氨基酸是谷氨酰胺和天冬酰胺,这一现象与Wang等^[5]^测得的结果一致。其中,草莓中谷氨酰胺是有机氮化合物主要的储存方式,并且在有机氮化合物的合成中发挥重要作用^[46,47]^。

**图4:**
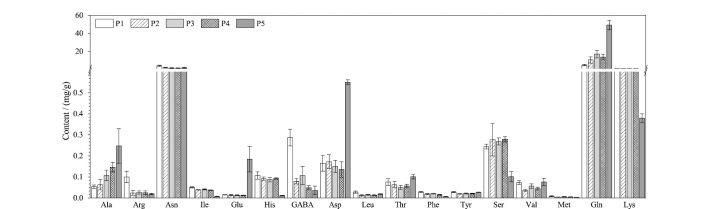
不同成熟时期草莓中游离氨基酸的含量(*n*=6)

通过对草莓不同成熟期各游离氨基酸的含量进行统计分析,比较了各成熟期氨基酸含量的差异。热图分析(图5a)结果中,以横坐标表示不同成熟时期的草莓,以纵坐标表示各氨基酸的浓度水平,不同的浓度水平通过不同颜色表示,其中红色表示氨基酸在草莓中含量高于平均值,蓝色表示氨基酸在草莓中含量低于平均值。采用偏最小二乘-判别分析(PLS-DA)模型建立得分图,对5个不同成熟时期的草莓进行区分。相关性系数(*R*^2^)表示模型的拟合性,*R*^2^≥0.7表示模型的解释能力较好;预测值(*Q*^2^)表示模型的预测效果,*Q*^2^≥0.5表示模型具有较好的预测能力。通过交叉验证评估PLS-DA模型^[48]^, *R*^2^、*Q*^2^和准确率分别为0.94、0.83和0.98,表明模型具有良好的可靠性和准确性。PLS-DA模型的得分图(图5b)表明,大绿期、大白期和片红期之间重叠较为严重,表明这3个时期在游离氨基酸成分的组成和含量上比较相近,而小绿期和全红期区分明显,表明这两个时期在游离氨基酸成分的组成和含量上存在较大差异。

**图5:**
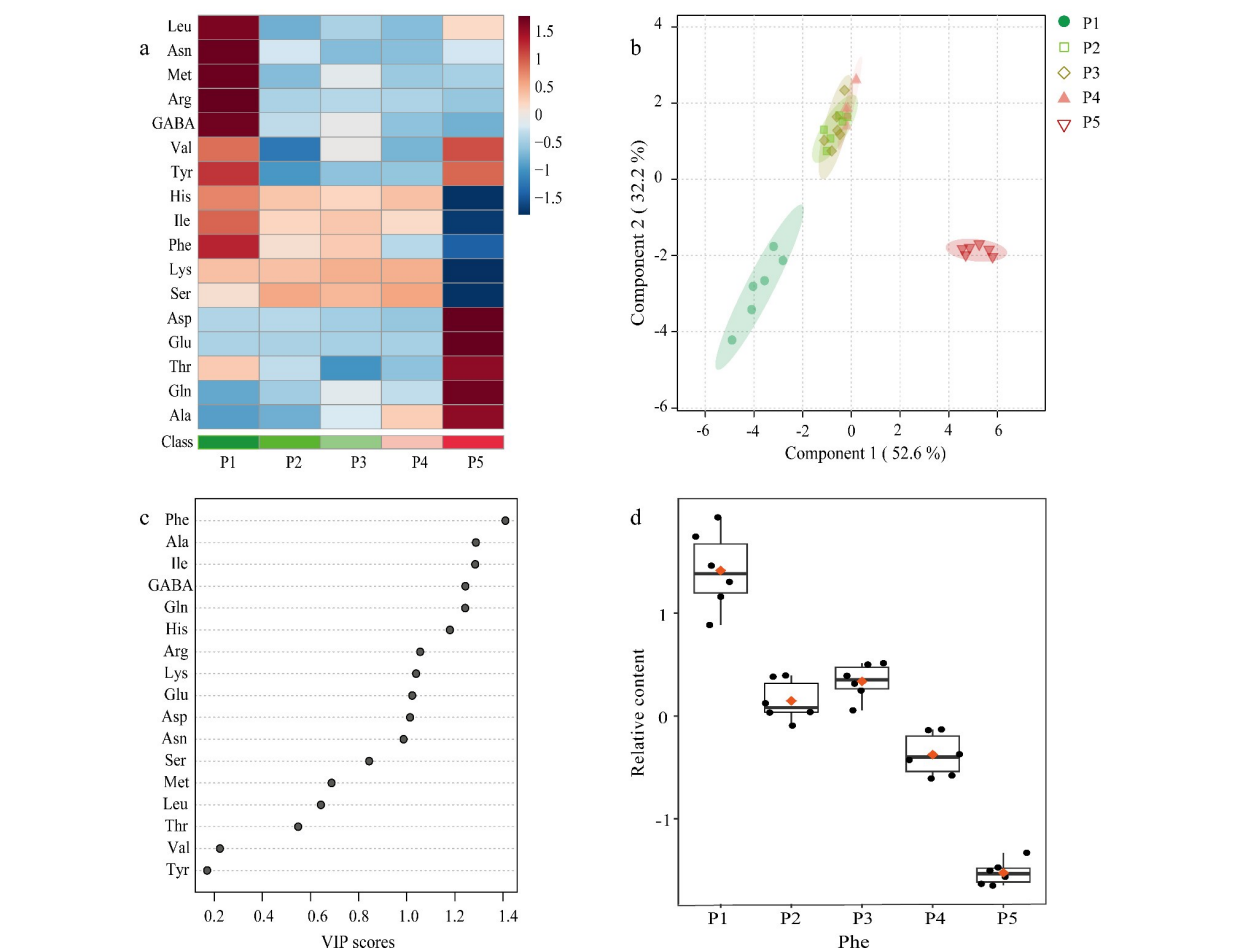
不同成熟时期草莓中氨基酸含量的统计分析

基于PLS-DA分类模型,采用变量投影重要性值(VIP)区分草莓5个不同成熟时期的潜在化学标志物(图5c), VIP值>1意味着该变量在模型中具有较高的重要性^[49]^。选择VIP值>1的10种氨基酸(丙氨酸、精氨酸、异亮氨酸、谷氨酸、组氨酸、4-氨基丁酸、天冬氨酸、苯丙氨酸、谷氨酰胺和赖氨酸)再进一步进行*t*检验,以VIP值>1和*P*<0.01为标准^[50]^,筛选出7种差异性氨基酸(苯丙氨酸、异亮氨酸、谷氨酰胺、4-氨基丁酸、精氨酸、谷氨酸和天冬氨酸),其中苯丙氨酸、异亮氨酸、4-氨基丁酸和精氨酸在草莓小绿期含量最高,可以将其作为小绿期区别于其他时期的差异性氨基酸;同样谷氨酰胺、谷氨酸和天冬氨酸也可以作为全红期区别于其他时期的差异性氨基酸。以VIP值最高的苯丙氨酸为例,如图5d所示,苯丙氨酸在不同成熟阶段含量分布差异较大,且随着草莓的不断成熟苯丙氨酸的含量也随之降低。同时,由图5d的箱线图结果可以看出,将不同成熟阶段的草莓样品各取6次平行测定,以苯丙氨酸为例,6次平行实验具有较好的重复性。

## 3 结论

本研究采用亲水相互作用液相色谱柱,建立了基于亲水相互作用的超高效液相色谱-串联质谱测定草莓样品中游离氨基酸含量的方法。该方法具有前处理简单、灵敏度高和重复性良好等优点。通过统计分析,比较了不同成熟时期草莓中游离氨基酸的含量差异,最终筛选出了7种具有显著差异的氨基酸。游离氨基酸含量的测定为草莓的营养研究提供了科学依据,该方法可推广应用于其他水果和蔬菜中游离氨基酸的组成分析、质量控制和代谢研究。
